# OCD Influences Evidence Accumulation During Decision Making in Males but Not Females During Perceptual and Value-Driven Choice

**DOI:** 10.3389/fpsyt.2021.687680

**Published:** 2021-07-29

**Authors:** Xiao Ma, Ashton Megli, Christopher Pittenger, Helen Pushkarskaya

**Affiliations:** ^1^Department of Psychiatry, School of Medicine, Yale University, New Haven, CT, United States; ^2^Department of Neuroscience, School of Medicine, Yale University, New Haven, CT, United States; ^3^Yale Child Study Center, School of Medicine, Yale University, New Haven, CT, United States

**Keywords:** obsessive compulsive disorder, evidence accumulation, drift diffusion model of choice, perceptual decisions, value-based decisions, gender differences

## Abstract

Individuals with obsessive-compulsive disorder (OCD) often have difficulty making decisions. Valuation and value-based judgements are particularly difficult. The mechanisms underlying these impairments are still poorly understood. Previous work has suggested that individuals with OCD require more information prior to making a choice during perceptual discrimination tasks. Little previous work has examined value-guided choice in OCD. Here we examined perceptual and value-based decision making in adults with OCD, using a novel task in which the two types of decision are tested in parallel using the same individually calibrated sets of visual stimuli (Perceptual and Value-based decision-making task, PVDM). Twenty-seven unmedicated participants with OCD (16 female) and thirty-one healthy controls (15 female) were tested. Data were analyzed using hierarchical drift-diffusion modeling (HDDM). Decision formation was altered in OCD, but differentially between genders: males with OCD, but not females, accumulated more information (i.e., were more cautious) and were less effective in evidence accumulation than age- and IQ-matched healthy males. Furthermore, males with OCD, but not females, were less likely than controls to adjust the process of evidence accumulation across decision contexts. These unexpectedly gender-dimorphic effects suggest that more attention should be paid to gender differences in studies of OCD, and of pathophysiology more broadly.

## Introduction

Decision making and information processing are aberrant in individuals with OCD. Indecisiveness, doubt, and impaired behavioral control are common; behavioral inflexibility has been suggested as a neurocognitive endophenotype (1–5). Deeper understanding of these deficits may provide better insights into the phenomenology and pathophysiology of the disorder and may thereby contribute to the development of new targets for therapeutic interventions. OCD is markedly heterogeneous. Careful characterization of individual variation in information processing and decision making may provide insight into this heterogeneity and ultimately contribute to individualized treatment selection.

Real-world decisions are not made instantaneously: evidence is accumulated over time until a decision is reached (6). Self-report measures and direct measures of behavior (e.g., choice accuracy and reaction time) provide limited insight into irregularities in evidence accumulation. Computational modeling of behavioral data, such as the Drift-Diffusion Model of choice (DDM), can better quantify individual variations in underlying decision formation processes and help to identify the corresponding neurobiology (7). The DDM approach is a powerful tool for examining individual differences in a process of decision formation, since even the small and medium effect size differences in observed behavioral measures (choice accuracy and reaction time) can correspond to larger effect sizes for differences in the latent decision parameters (8). Thus, laboratory samples of a relatively modest size (total *N* ~60) are well-powered to detect between-group effects of interest. Using Hierarchical Bayesian estimation of DDM parameters [HDDM; (9)] further improves power of such analyses (10). Thus, parameters derived using HDDM are increasingly used in decision science, both in studies of basic mechanisms and in studies of decision making in clinical populations (11).

**Figure 1:**
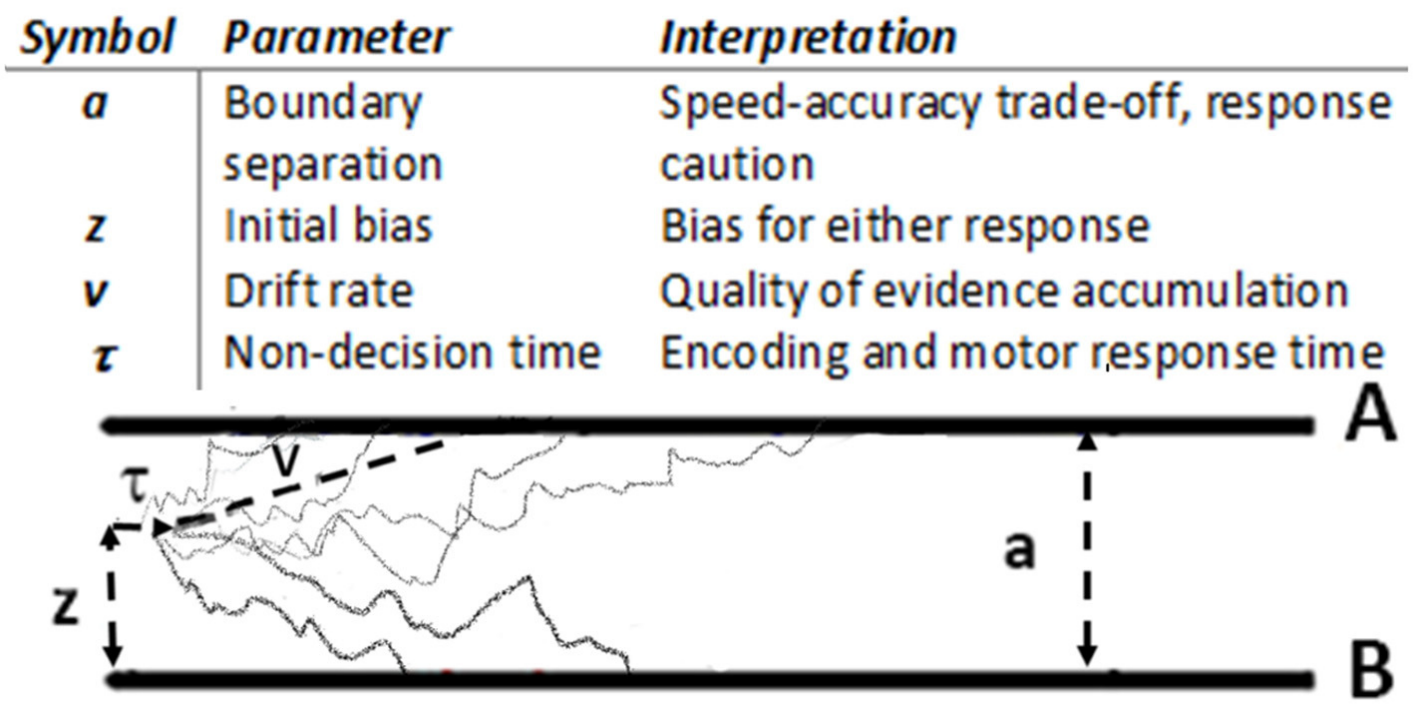
Drift diffusion model (6): main parameters and graphical representation. During stimulus presentation, evidence is accumulated with an average rate *v* until reaching the Boundary A or B.

The DDM framework [Figure 1 (6)] suggests that a choice is made only after accrued evidence in favor of one of the available options crosses a critical threshold (termed the decision threshold or boundary separation). Lower decision thresholds produce less accurate, more impulsive choices; higher decision thresholds lead to more accurate, more cautious choices. The time it takes to make a decision is determined both by this threshold and by the rate of evidence accumulation, termed the drift rate. The drift rate reflects effectiveness of evidence accumulation during decision formation, or the signal-to-noise ratio of the evidence accumulation process; it has been shown to positively correlate with general cognitive abilities [e.g., IQ, (12)]. Optimal decision making requires adjustment of decision thresholds and drift rates in response to current task demands. For instance, more difficult tasks (e.g., “which is sweeter, Pepsi or Coke?”) require more evidence to be accumulated before a decision is made (higher decision threshold) and may slow down the process of accumulation of such evidence (reduce the drift rate). On the other hand, easier choices (e.g., “what is hotter, ice-cream or hot tea?”) can be made with very little additional evidence and can be processed very effectively (i.e., lower thresholds and higher drift rates).

Adjustments of DDM parameters in response to task demands vary among individuals. For instance, on four different tasks [a signal detection task (13), letter discrimination (14), masked brightness discrimination (15), and recognition memory (16)], when instructed to make choices as quickly as possible, college students were more willing to sacrifice accuracy for speed (i.e., to reduce decision thresholds) than participants who were older than 60 (16). During the random dots motion task (RDM), individuals with OCD increased their decision thresholds in response to increased task difficulty significantly more than did age-matched healthy individuals (17). This may correspond to the indecision and doubt often seen in OCD, especially during difficult tasks. When a monetary incentive penalizing slow responses was introduced, individuals with OCD decreased the decision threshold more than healthy individuals, accumulating less evidence during easier choices (17). This may reflect heightened sensitivity to potential negative outcomes [i.e., loss aversion (18)].

Several studies have used the DDM framework to examine perceptual decisions in individuals with OCD (17, 19, 20). Perceptual decisions involve the integration of sensory evidence to produce a categorical choice between options [e.g., “one item is larger than the other one,” or “more dots are moving to the left than to the right” (16)]. Recent evidence suggests that value-based subjective judgements (e.g., “I like oranges more than apples”) are also impaired in OCD. For instance, individuals with OCD have been shown to be more inconsistent in their value-based choices (i.e., to prefer option A to option B during some trials and prefer option B to option A during other trials within the same gambling task) and to choose objectively suboptimal options more often than healthy individuals [e.g., choose a 50–50 gamble of wining $5 or nothing over a certain payoff of $5; Pushkarskaya, Tolin (21)]. It has been suggested that impaired valuation in OCD may explain a close link between OCD symptoms and anhedonic tendencies, independent of general depression (22, 23).

Perceptual and value-base judgements are typically independent. For instance, one may equally like small kiwi and large watermelon, or prefer one black dress to another. Studies that characterize evidence accumulation during value-based judgement using DDM in the general population are sparse (24–26); but there is some evidence that healthy individuals (25, 27) process information more efficiently but respond more cautiously during perceptual than during value-based choice. Value-based choice has not been examined using DDM in OCD, or indeed in any other forms of psychopathology. Examining how DDM parameters (e.g., decision thresholds) adjust in response to task demands across contexts (e.g., not only easy vs. difficult choices, but also perceptual vs. value-based choices) may help characterizing OCD-associated impairments in decision formation more broadly.

OCD-associated impairments in decision making, both under certainty and uncertainty, have been assessed using a broad range of self-report instruments and behavioral tasks (28). An important consideration in all such studies is that OCD is markedly heterogeneous in specific symptoms (29), the motivation that drives these symptoms [incompleteness and harm avoidance (30)], comorbidity (31), and natural history (32). Some of this heterogeneity may be attributable to sex/gender (33–35). Reviews of the literature describing sexual dimorphism in OCD have progressed over the decades from dismissing the possibility of gender effects in OCD (36) to acknowledging growing evidence (29, 35, 37, 38). OCD is more common among males in childhood, but among females in adolescence and adulthood (39). Females with OCD tend to report higher depression and anxiety (35), to exhibit more contamination/cleaning symptoms, and to have greater comorbidity with eating and impulse-control disorders (38). Research on deficits in executive functioning in OCD (40–44) has not systematically examined gender differences; most laboratory studies are not adequately powered for such analyses. However, a recent metanalysis found that the proportion of females in individual studies correlated with effect sizes of some neuropsychological impairments in OCD, suggesting the possibility of a sex/gender effect (45). Specifically, in samples with more female participants, the OCD group had worse performance on set shifting and working memory tasks.

In this study, to directly test OCD-associated impairments in decision formation across contexts for sexual dimorphism, we recruited gender balanced samples of unmedicated individuals with OCD and of healthy individuals to complete a novel decision-making task, the Perceptual and Value-based Decision Making (PVDM) task. We analyzed these data using HDDM to contrast decision formation across contexts (perceptual vs. value-based, easy vs. difficult) in individuals with OCD and healthy controls, and to test whether OCD-associated impairments in decision formation across contexts are modulated by gender.

## Materials and Methods

### Participants

All procedures were approved by the Yale University Human Investigation Committee. All participants provided written informed consent and completed a demographic questionnaire and the Kaufman Brief Intelligence Test (46). All participants completed the Perceptual and Value-based Decision-Making task (PVDM, detailed below) and were compensated for their time.

A priori power analysis indicated that, given anticipated large effect sizes [Cohen's *f* > 0.4 (8)], to detect differences between 4 groups using ANCOVA while controlling for age and IQ with *p* < 0.05 and power equal to 0.8, we needed a total sample of *N* > 52 (47). We used HDDM to further improve the power of planned analyses (10).

Twenty-nine adults with OCD (17 females; age range = 18–62 years, mean = 31 ± 11 SD), unmedicated for at least 8 weeks, and thirty-two healthy adults (16 females; age range = 18–59 years, mean = 30 ± 11 SD) were recruited through the Yale OCD Research Clinic (ocd.yale.edu). Diagnoses were established by doctoral-level clinicians and confirmed using the Mini International Neuropsychiatric Interview 7.0.2 [MINI (48)]. Clinically significant OCD symptoms were defined as Y-BOCS ≥ 16. OCD was the primary clinical diagnosis in all twenty-nine individuals with OCD; fifteen of them also reported clinically significant comorbidities, which included panic disorder (7), depression (6), social phobia (4), agoraphobia (3), PTSD (2), and GAD (2). None of our study participants meet criteria for comorbid impulse control disorder. Severity of obsessions and compulsions was assessed using the Yale-Brown Obsessive-Compulsive Scale [Y-BOCS (49, 50)] and severity of depression using Beck Depression Inventory – II scale [BDI - II (51)]. OCD symptoms were also assessed dimensionally, using Obsessive-Compulsive Inventory revised [OCI-R, (52)], Dimensional Obsessive-Compulsive Scale [DOCS, (53)], and the Obsessive-Compulsive Trait Core Dimensions Questionnaire [OC-TCDQ, (54)]. These assessments were administered within 1 week of behavioral testing. Exclusion criteria included IQ <70, current severe major depression (BDI-II ≥ 29), a primary psychotic disorder, autism, moderate or severe substance use disorder within the past 6 months, and poor visual acuity (after correction).

### Perceptual and Value-Based Decision-Making Task (PVDM)

In a preliminary study, 20 unscreened individuals, recruited from the general population in the New Haven area using flyers, rated 200 grayscale images (judged to be affectively neutral by a principal investigator) on a sliding scale from “1” (= Do not like) to “7” (= Like very much). Participants were also asked to classify all images as “neutral” or “emotional.” All images classified as “emotional” by ≥ 2 people were excluded. For all remaining images, the average liking rating was calculated, as was the average grayscale density (i.e., “blackness”). One hundred-twenty images were selected with a uniform distribution of “liking” and “blackness” ratings.

The PVDM task consists of two interleaved experimental conditions, perceptual (PDM) and value-based decision making (VDM), presented in 3 phases, one after the other, in a single session. Phase I was Rate I, Phase II was Choices, and Phase II was Rate II, as detailed below. The same images were used in both experimental conditions (PDM and VDM), allowing us to control for various potential confounds.

**Figure 2:**
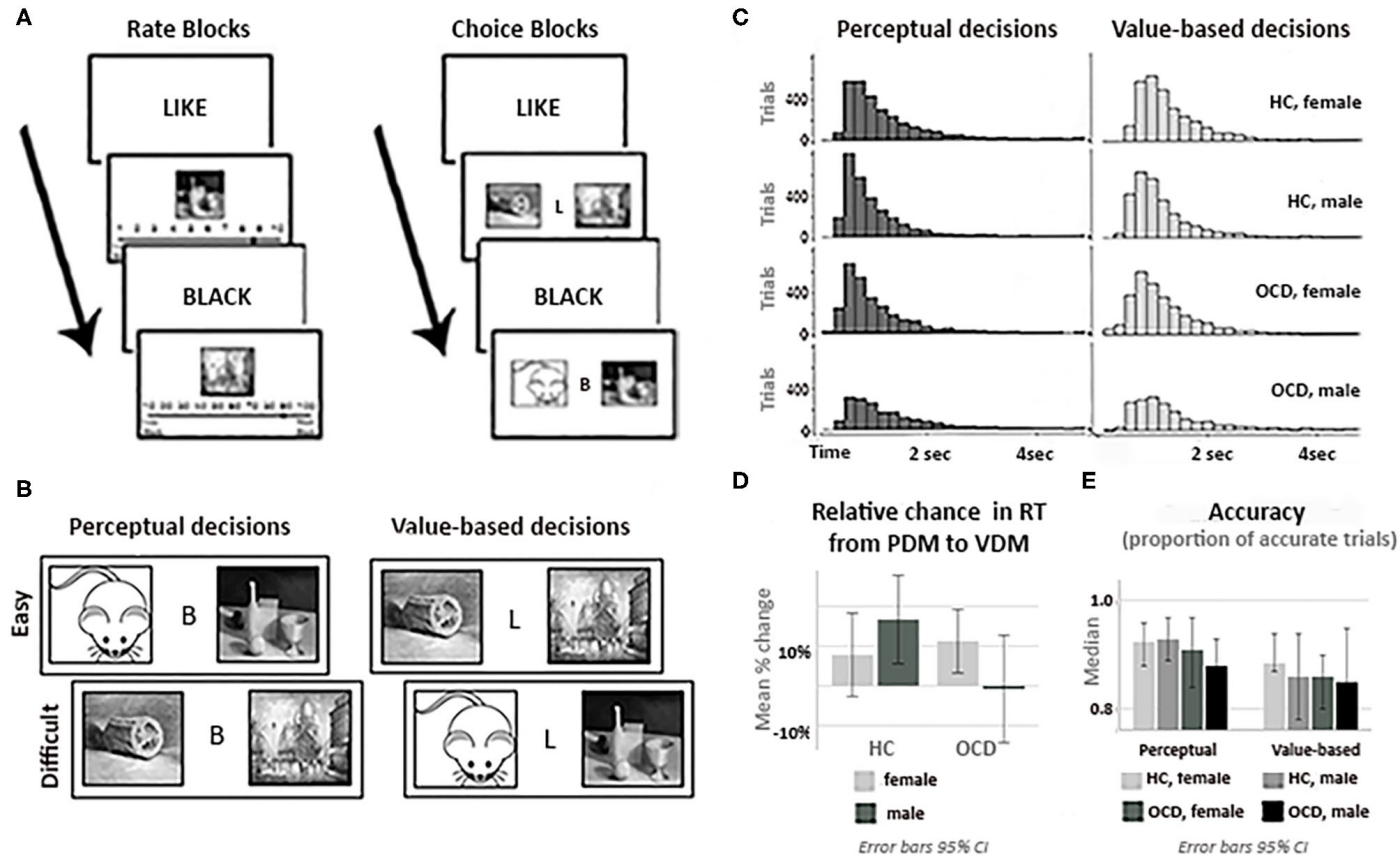
Experimental tasks and behavioral analyses. **(A)** Trial structure: rate (Phase I) and choice (Phase II) phases. **(B)** Task stimuli: the same neutral images were used for perceptual (PDM) and value-based (VDM) trials. **(C)** RT histograms for PDM and VDM trials for all participants groups. **(D)** Relative change in the mean reaction time (RT) from PDM to VDM choices by diagnostic group and gender. **(E)** The median accuracy (proportion of trials with accurate responses, based on rate II) for all participants groups during PDM and VDM trials. Accuracy of choices during PDM or during VDM trials, as well as the percentage change in accuracy from PDM to VDM trials, did not differ significantly across the diagnostic or gender groups. Error bars in **(D,E)**. are 95% CI.

During Phase I (Rating I), participants provide individual perceptual and value-based ratings (i.e., blackness and liking) of the 120 grayscale images, presented one at time in the middle of the screen in a pseudorandom order (Figure 2A). For perceptual ratings, participants were instructed to estimate “what portion of the image (in percent) is covered by black ink, assuming that all white is 0%, all black is 100%, and all evenly gray is 50%,” on a scale from 10 to 90% in steps of 10%. For value-based ratings, participants were instructed to indicate “how much do you like this painting” with a sliding scale from “1” (= Do not like) to “9” (= Like very much). This phase was untimed; the typical time required to complete it was about 10 min.

In Phase II (Choices, Figure 2A), images were presented in pairs, and the participant was instructed to pick one. Before the beginning of Phase 2, an algorithm selected a subset of the 120 images, providing balanced image pairs, based on the subject's own ratings, for both PDM and VDM trials. The difficulty of discriminating between pairs of images was calculated from ratings of images in Phase 1. The difficulty of choices was assessed as follows. Let three images, Image 1, Image 2, and Image 3, during Rate 1 be judged as being 40% black, 30% black and 90% black, respectively. Then deciding which image has more black color would be easier given a pair of Image 1 (40% black) and Image 3 (90% black) than given a pair of Image 1 (40% black) and Image 2 (30% black). During PDM choice trials, we classified a choice between Image 1 and Image 2 as difficult (10% separation) and a choice between Image 1 and Image 3 as easy (50% separation). Similarly, let three images, Image 1, Image 2, and Image 3, be valued on the “likeness” scale as 4, 3, and 9, respectively. During VDM choice trials, we classified a choice between Image 1 and Image 2 as difficult (1 point separation), and a choice between Image 1 and Image 3 as easy (5 points separation).

One hundred unique pairs of images were generated for each condition, 25 for each level of difficulty (“proportion black” separation of 10, 20, 40, or 50%; “liking” separation of 1, 2, 4, or 5). Choices between similarly rated images (10 or 20% difference for PDM; 1 or 2 for VDM) were defined as difficult choices; choices between more widely separated images (40 or 50% for PDM; 4 or 5 for VDM) were defined as easy choices (Figure 2B). Each pair of images was displayed twice, for a total of 400 trials. On each choice trial, participants were asked to select one of the two images by pressing a button “1” for an image on the left and “2” for an image on the right, with no time limit. Choice trials were grouped in four blocks (two blocks of PDM trials and two blocks of VDM trials, 100 trials each; order of blocks is counterbalanced across subjects). Each block started with screen that announces what type of choice are given during this block (“Please choose an image that you like more” for VDM trials, or “Please choose the image that is darker” for PDM trials). On average, total time required for this phase was about 20 min.

Importantly, the selection of images for Phase II Choices was such that “blackness” of images did not correlate with “likeness” of these images for an individual subject. This was to make sure that value-based characteristics of the images (i.e., how much a participant liked or disliked the image) did not systematically affect perceptual characteristics of the images (i.e., how dark the image was).

Phase III (Rating II) was identical to Phase I; these data are used to examine the stability of ratings.

During the experiment, participants sat ~80 cm away from the 22-inch monitor screen (resolution 1,680 ×1,050 pixels); all images were of the same size during all phases (Rate I, Choice, and Rate II): length ~0.30°; height ~0.45° (recall that during Choice phase two images were presented on the screen).

Thus, PVDM allows characterizing and contrasting perceptual and value-based judgments using the same set of stimuli. During PDM trials, participants were asked to make binary choices based on accumulated sensory evidence; these choices reflect perceptual judgements. During VDM trials, participants were asked to make binary choices based on their subjective valuation of each image; these choices reflected their subjective value-based judgements. The selection of images for Phase II Choices was such that “blackness” of images did not correlate with “likeness” of these images.

The task was explained to participants at the beginning of the experiment. Next, participants complete 3 practice PDM and 3 VDM trials from each phase, and they were given an opportunity to ask questions. During practice, PDM trials, on which there is an objectively correct answer, feedback was provided. After participants had an opportunity to ask questions and to discuss this feedback and confirmed that they understood the task, they proceeded to the experiment, starting with Phase I. To incentivize accurate choices, participants were told that at the end of the experiment, one trial from the Choices phase would be randomly selected. If a VDM trial was selected, participants received a copy of the selected image in a 4” × 4” frame. If a PDM trial was selected and they chose the objectively correct response, they received $5 in addition to the base participation fee of $40.

### Computational Modeling

#### Data Pre-processing

Choice data and reaction time data were recorded for each participant and preprocessed as described below for fitting to the drift-diffusion model of two-alternative forced-choice decision-making tasks (6). DDM fitting requires two types of input data: response time and accuracy of each choice.

Data from 61 subjects (12 males with OCD, 17 females with OCD, 16 healthy males, 16 healthy females) was examined for random and careless responses. First, we excluded subjects who rated all images during rate Like phase only as “1” (= Do not like at al), “5”(Neutral), or “9” (= Like very much). Second, for each image, we compared ratings from Rate I and Rate II phases. While some variations in ratings between two phases are expected; significant changes are indicative of careless or unreliable ratings. Thus, if ratings of the same image during Rate I and Rate II differed in more than 4 points, we removed VDM trials that used this image for this subject. If more than 15% of trials were excluded for the subject's data, data from this subject was excluded from the analysis as unreliable. Based on these two criteria, three subjects were excluded from the analysis (1 healthy female, 1 female with OCD, and 1 male with OCD). Thus, data from 58 subjects (11 males with OCD, 16 females with OCD, 16 healthy males, 15 healthy females) were included in the next step of analyses.

Next, since DDM fitting is sensitive to outliers (55), we examined data for short and long outliers. First, all trials with RT > 6 s or <0.2 s were discarded (56). Next, for each individual subject we excluded trials that were classified as extreme outliers (RT < mean - 3 SD or RT > mean + 3 SD).

Accuracy in PDM trials was defined with respect to the objective “blackness” of the images. Accuracy was “1” for trials when participant chose the image with the higher density and “0” otherwise.

Accuracy in VDM trials was defined based on the individual subjective ratings provided during the Rate I and Rate II task phases. It was not uncommon for individuals to change their valuation of some images after being forced to make a choice between them [the “choice-induced preferences effect” (57)]. However, large changes in the ratings of an image within a short period of time may indicate careless responses. Thus, for each participant we excluded trials containing images that were rated highly inconsistently during Rate I and Rate II (difference between two ratings >4 points). For the remaining images, we defined three measures of subjective valuation: (1) based on responses from Rate I, (2) based on responses from Rate II, and (3) based on the average of these two ratings [i.e., mean (Rate I, Rate II)]. Accuracy on each choice was determined using each of these measures. As detailed below, DDM was computed using each of these accuracy measures, to determine which gave the best model fit.

#### Descriptive Analysis of Behavioral Data

To assess the effect of decision context on reaction time (RT) and accuracy, we computed the relative change in average RT for each subject [e.g., (mean RTi during value-based choices – mean RTi during perceptual choices)/mean RTi during perceptual choices for all i = 1,…,58 subjects in our sample] and accuracy rates [e.g., (mean Accuracyi during value-based choices – mean Accuracyi during perceptual choices)/mean Accuracyi during perceptual choices for all i = 1,…,58 subjects] and used one-sample *t*-test for normally distributed variables (i.e., Shapiro-Wilk test *p*-value is > 0.05) or non-parametric one-sample Wilcoxon signed-rank test for variables that were not normally distributed to test the resulting values against a mean (or median) of 0, using SPSS statistics (v26, IBM, New York, USA). The significance threshold was set to 0.05, adjusting for multiple comparisons using the Bonferroni method. To examine whether OCD diagnosis and/or gender modulate the effect of the decision context on reaction times and accuracy, we employed univariate 2 × 2 ANOVA using SPSS statistics for normally distributed variables and nonparametric ANOVA using R (“aligned.rank.transform” routine) for variables that were not normally distributed.

#### Model Fitting

Preprocessed response time and accuracy data were analyzed using hierarchical Bayesian parameter estimation in the Drift Diffusion Model [HDDM (9)]. We particularly focus on two DDM parameters – decision threshold, a, and the drift rate, v, (Table 1, Figure 1) – and on how these parameters respond to task demands (perceptual vs. value-based decisions, easy vs. difficult choices), and how these adjustments are modulated by the OCD diagnosis and gender.

**Table 1:** Demographic and clinical characteristics of the study participants.

	**Healthy individuals**			**Individuals with OCD**			**OCD vs. HC**	
	**Males**	**Females**	**Pooled**	**Males**	**Females**	**Pooled**	***t* (58)**	***p*-value**
	**Mean (SD)**							
**Demographics**								
Age	29.6 (10.1)	27.5 (11.4)	28.5 (10.6)	32.8 (13)	29.1 (9.5)	30.6 (11)	−0.7	0.5
IQ	112 (13.3)	114 (12.2)	**113 (12.7)**	107 (14)	105 (13.7)	**106 (13.7)**	2	**0.03**†
							***t*** **(55)**	
**Clinical symptoms**								
YBOCS	–	–	–	21.8 (3.5)	24.6 (5.5)	23.5 (4.9)	–	–
BDI – II	6.9 (7.3)	4.2 (4.0)	**5.6 (6.0)**	10.8 (12.8)	13.3 (12.9)	**12.4 (12.6)**	−2	**0.2**
DOCS	7.9 (9.4)	5.8 (5.6)	**6.9 (7.8)**	22.0 (9.4)	27.0 (11.5)	**25.1 (10.8)**	−7	** <0.01**
OCI-R	6.9 (9.1)	5.9 (5.4)	**6.4 (7.4)**	**18.5 (7.5)**	27.7 (10.1)^*^	**24.0 (10.1)**	−7	** <0.01**

*In bold are significant differences between groups.*

* *Females with OCD scored significantly higher than males with OCD on OCI-R (p =0.03). †Healthy participants on average scored ~8 point higher on IQ test than OCD participants. YBOCS was not evaluated in healthy controls*.

To improve the quality of parameter estimation, we employed the basic 4-parameter model (58) and allowed these parameters to vary across trial types (PDM vs. VDM) and choice difficulty (easy vs. difficult). Next, to examine effects of interest, we allowed three of the parameters (the decision threshold, the drift rate, and the non-response time) to depend on the subject's diagnosis (Dx: OCD or HC) and gender. Finally, we included covariates that have been shown to affect the decision threshold and the drift rate in prior studies and that potentially could confound our estimates of effects of OCD diagnosis and gender. This approach produces the following models:

Model 0: a ~ trial type, difficulty; v ~ trial type, difficulty; τ ~ trial type, difficulty; z;
Model 1: a ~ trial type, difficulty, Dx; v ~ trial type, difficulty, Dx; τ ~ trial type, difficulty, Dx; z;
Model 2: a ~ trial type, difficulty, gender; v ~ trial type, difficulty, gender; τ ~ trial type, difficulty, gender; z;
Model 3: a ~ trial type, difficulty, Dx, gender; v ~ trial type, difficulty, Dx, gender; τ ~ trial type, difficulty, Dx, gender; z;

Next, in Models 4–6, we included age and IQ as covariates, since they have been previously to affect the decision threshold and the drift rate and excluding them could potentially confound our results (12, 16). We did not include age, IQ, and other variables as covariate for τ in Models 4–8 since we did not have a priori hypothesis and to avoid overfitting the model.

*Model 4: a* ~ trial type, choice difficulty, **Dx, gender, age**; *v* ~ trial type, choice difficulty, **Dx, gender, age**; τ ~ trial type, choice difficulty, **Dx, gender**; *z*;
*Model 5: a* ~ trial type, choice difficulty, **Dx, gender, IQ**; *v* ~ trial type, choice difficulty, **Dx, gender, IQ**; τ ~ trial type, choice difficulty, **Dx, gender**; *z*;
*Model 6: a* ~ trial type, choice difficulty, **Dx, gender, age, IQ**; *v* ~ trial type, choice difficulty, **Dx, gender, age, IQ**; τ ~ trial type, choice difficulty, **Dx, gender**; *z*.

In Model 7, we examined whether including severity of depression as covariate changes our estimates of effects of OCD diagnosis and gender on the decision threshold and the drift rate. Several prior studies reported that depression may affect a process of evidence accumulation, specifically, by making the decision thresholds wider (59, 60). Since individuals with OCD tend to report more of depressive symptoms than healthy individuals, not including severity of depression may potentially confound estimates of the effect of OCD diagnosis.

*Model 7: a* ~ trial type, choice difficulty, **Dx, gender, age, IQ, BDI**; *v* ~ trial type, choice difficulty, **Dx, gender, age, IQ, BDI**; τ ~ trial type, choice difficulty, **Dx, gender**; *z*.

Finally, in Model 8, we examined whether including self-reported impulsivity [measured by Barat Impulsivity Scale, BIS-11 (61)], as covariate changes our estimates of effects of OCD diagnosis and gender on the decision threshold and the drift rate. Note that impulsivity is a complex, multifaceted concept, and BIS-11 and decision threshold are likely to quantify different components of impulsivity (62). Still, not including a measure of impulsivity may confound estimates of the effect of OCD diagnosis on DDM parameters.

*Model 8: a* ~ trial type, choice difficulty, **Dx, gender, age, IQ, BIS**; *v* ~ trial type, choice difficulty, **Dx, gender**; τ ~ trial type, choice difficulty, **Dx, gender**; *z*.

Selection of the final model was based on deviance information criteria [DIC (63)] and on the comparison of posterior predictive probability density plots with the data-based normalized RT distribution for each condition.

## Results

### Clinical and Demographic Characteristics

Data from 58 participants, HC males (*N* = 16), HC females (*N* = 15), OCD males (*N* = 11), and OCD females (*N* = 16), are reported here. The four groups did not differ in age (*p* = 0.67), education (*p* = 0.6), or income (*p* = 0.5, see Table 1 and Supplementary Table 1), which suggests that our efforts to match four groups of interest on socio-demographic characteristics was largely successful. However, OCD participants scored on average ~7 point lower than healthy participants on IQ (*p* = 0.03).

Three participants did not complete clinical self-report scales (1 healthy male, 1 healthy female, and 1 male with OCD). Analysis of the data from the remaining fifty-five participants revealed that, consistent with clinical diagnoses, individuals with OCD scored higher than healthy individuals on OCD symptom severity scales and on depression (see Table 1, Supplementary Table 1). Four groups of interest did not differ significantly on BIS-11, even though males with OCD reported qualitatively lower scores than females with OCD (see Supplementary Table 1). Severity of OCD symptoms was somewhat greater in females with OCD than in males with OCD, though the effect was modest and significant only for one of three measures (OCI-R and not for YBOCS or DOCS, see Table 1); severity of comorbid depression did not differ between genders. Different symptom dimensions, as measured by OCI-R, were all similarly slightly elevated in females (see Supplementary Table 1).

Note that the OCI-R and DOCS scores for males with OCD are relatively low for an OCD sample, even though Y-BOCS scores for this group were at the expected level (recall that all OCD participants were required to have Y-BOCS ≥ 16). It is possible that our males with OCD participants somewhat underreported their symptoms when completed self-report scales. But examining this possibility is beyond the scope of this study.

### Descriptive Statistics of Behavioral Data

Mean RT across decision contexts was distributed normally in our sample [Kolmogorov-Smirnov statistics (58) = 0.087, *p* = 0.20], but mean accuracy was not [Kolmogorov-Smirnov statistics (58) = 0.147, *p* =0.003]. Thus, RT was analyzed using *t*-test and 2 × 2 ANOVA, while accuracy was analyzed using non-parametric one-sample Wilcoxon signed-rank test and nonparametric 2 × 2 ANOVA (see Methods).

As expected, in both PDM and VDM trials, it took longer for participants to choose between images with close ratings (more difficult choices) than between images with more widely separated ratings (easier choices). For PDM trials, mean RT increase was 50.0% ± 32 SD [t (57) = 11.87, *p* < 0.001], and for VDM trials it was 27.6% ± 22 SD [t (57) = 9.52, *p* < 0.001]. VDM trials on average had higher RT than PDM trials [mean RT increase: 8.27% ± 21 SD, *t* (57) = 2.98, *p* = 0.004]. The main effect of diagnosis on this difference was not significant [HC: mean ΔRT = 11.7% ± 3.8%, OCD: mean ΔRT = 3.5% ± 4.1%, *F*_(1, 54)_ =1.16 *p* = 0.48]; neither was the gender x diagnosis interaction, even though this change was qualitatively lower in males with OCD [HC male: mean ΔRT = 15.6% ± 21 SD; HC female: mean ΔRT = 7.8% ± 22 SD; OCD male: mean ΔRT = −0.2% ± 22 SD; OCD female: mean ΔRT = 7.2% ± 19 SD, *F*_(1, 54)_ = 1.84 *p* = 0.18].

Also, as expected, in PDM, accuracy was lower during difficult choices than it was during easy choices; the median accuracy decrease was 8.2% ± 7.9 SD [z (58) = 154, *p* = 0.001]. Accuracy for VDM trials has not significantly changed with trial difficulty significantly across types of trials; during difficult trials it was lower by 10% ± 8.9 SD [z (57) = 0.086, *p* = 0.20]. Accuracy was higher on PDM trial than on VDM trials; the median accuracy increase was 2.0% ± 14 SD [z (58) = 197, *p* < 0.001].

The main effect of OCD diagnosis on RT was not significant [*F*_(1, 54)_ = 0.41 *p* = 0.64]. However, the gender x diagnosis interaction was significant [*F*_(1, 54)_ = 4.41 *p* = 0.04]. Males with OCD took longer to make decisions and healthy males were faster than other groups [HC male: mean RT = 1.20 ± 0.08 sec; HC female: mean RT = 1.41 ± 0.07 sec; OCD male: mean RT = 1.56 ± 0.16 sec; OCD female: mean RT = 1.33 ± 0.11 sec, see Figures 2C,D]. This suggests that the effect of OCD on decision making during perceptual and value-based choice is not homogeneous across diagnostic and gender groups.

Further examination revealed that in healthy males and females, and in females with OCD, RT significantly changed from VDM to PDM (*p* = 0.02) and from easy to difficult choices (*p* < 0.001).

Three measures of accuracy in VDM (based on Rate I, Rate II, and the average of Rate I and Rate II) strongly correlated (from r Rate I vs. Rate II = 0.91 *p* < 0.001 to r Rate I vs. mean Rate I,II = 0.97 *p* < 0.001). Accuracy on VDM trials on average was lower relative to PDM trials [estimated mean Accuracy decrease was between 2% ± 10 SD (based on Rate II) and 10% ± 9 SD (based on Rate I), *z* (57) <299, p corrected <0.01]. Neither the main effect of diagnosis [*F*_(1, 55)_ <1.02 *p* > 0.31] nor the gender x diagnosis interaction were significant [*F*_(1, 55)_ <1.49 *p* > 0.22], see Figure 2E.

### Computational Modeling

#### Model Selection

First, we examine which measure of accuracy of choices [based on Rate I, Rate II, or mean (Rate I, Rate II)] provided the best fit to the data. In this analysis we employed Model 0 (see Methods). Using accuracy based on Rate II significantly improved model evidence compared to the other 2 models [DICRateI = 43,433, DICRateII = 41,092, DICmean (RateI, RateII) = 41,920]; thus, in all subsequent analyses we used Rate II measures to determine accuracy in VDM trials.

Next, we computed DIC and examined posterior predictive probability density plots check (PPC) on Models 1–3 (see Methods). PPC revealed a good fit for all three models (see Supplementary Materials 2). Model 3, which includes both OCD and gender as between-subject factors, demonstrated similar fit (DICDx = 41,090, DICGender = 41,090, DICDx x Gender = 41,090). However, distributions of reaction time differed across diagnostic and gender groups (see Figure 2), and, consequently, the PPC favored Model 3.

Next, we examined the effect of including covariates on both model fit and parameter estimates.

Including both age and IQ as covariates did not further improve DIC (Model 4: DICDx x Gender, age = 42,056, Model 5: DICDx x Gender, IQ = 42,050, Model 6: DICDx x Gender, age, IQ = 42,049). In Model 4, age correlated negatively with the drift rate (95% CI: −0.12, −0.0045) but not with the threshold (95% CI: −0.054, 0.081); in Model 5, IQ correlated positively with the threshold (95% CI: 0.12, 0.25) and the drift rate (95% CI: 0.084, 0.20). In Model 6, only correlations of IQ with DDM parameters remained significant, indicating that IQ influences these parameters more strongly than age. OCD patients had ~7 points lower mean IQ; thus, controlling for IQ is important in our analyses. Age was qualitative (but not significantly) lower in healthy females and higher in males with OCD; thus the choice was made to keep age as a covariate in the model. Effects of OCD and Gender on parameters of interest were qualitatively similar in Models 4–6.

A subset of our study participants (*N* = 55) completed self-reported measures of severity of depression (BDI-II) and impulsivity (BIS-11). In this subset of subjects, Model 6 (DICDx x Gender, age, IQ = 37,293) performed better than Model 7 (DICDx x Gender, age, IQ, BDI = 38,213); BDI-II did not correlate significantly with DDM parameters (decision threshold 95% CI: −0.022, 0.13; drift rate 95% CI: −0.071, 0.067). Including BIS also did not improved DIC as compared to Model 6 (Model 8: DICDx x Gender, age, IQ, BIS = 38,211); BIS did not correlate significantly with the decision threshold (95% CI: −0.059, 0.12). Effects of OCD and Gender on parameters of interest in Models 7&8 were qualitatively similar to those seen in Models 6. Thus, Model 6 was selected as optimal for detailed analysis.

#### Effect of Diagnosis on Evidence Accumulation

Healthy males in our sample were more effective in processing of perceptual information than healthy females (i.e., had higher drifts rates) during easy choices [*posterior* p (v_HC, male_ > v_HC, female_) > 0.95] and during difficult choices [*posterior* p (v_HC, male_ > v_HC, female_) > 0.90]; they also collected less information prior to making a choice during difficult perceptual decisions [i.e., had lower decision thresholds, *posterior* p (a_HC, female_ > a_HC, male_) > 0.90], implying a trend toward higher reflection impulsivity [i.e., reduced amount of information gathered before taking a decision (64)] in healthy males in our sample.

**Figure 3:**
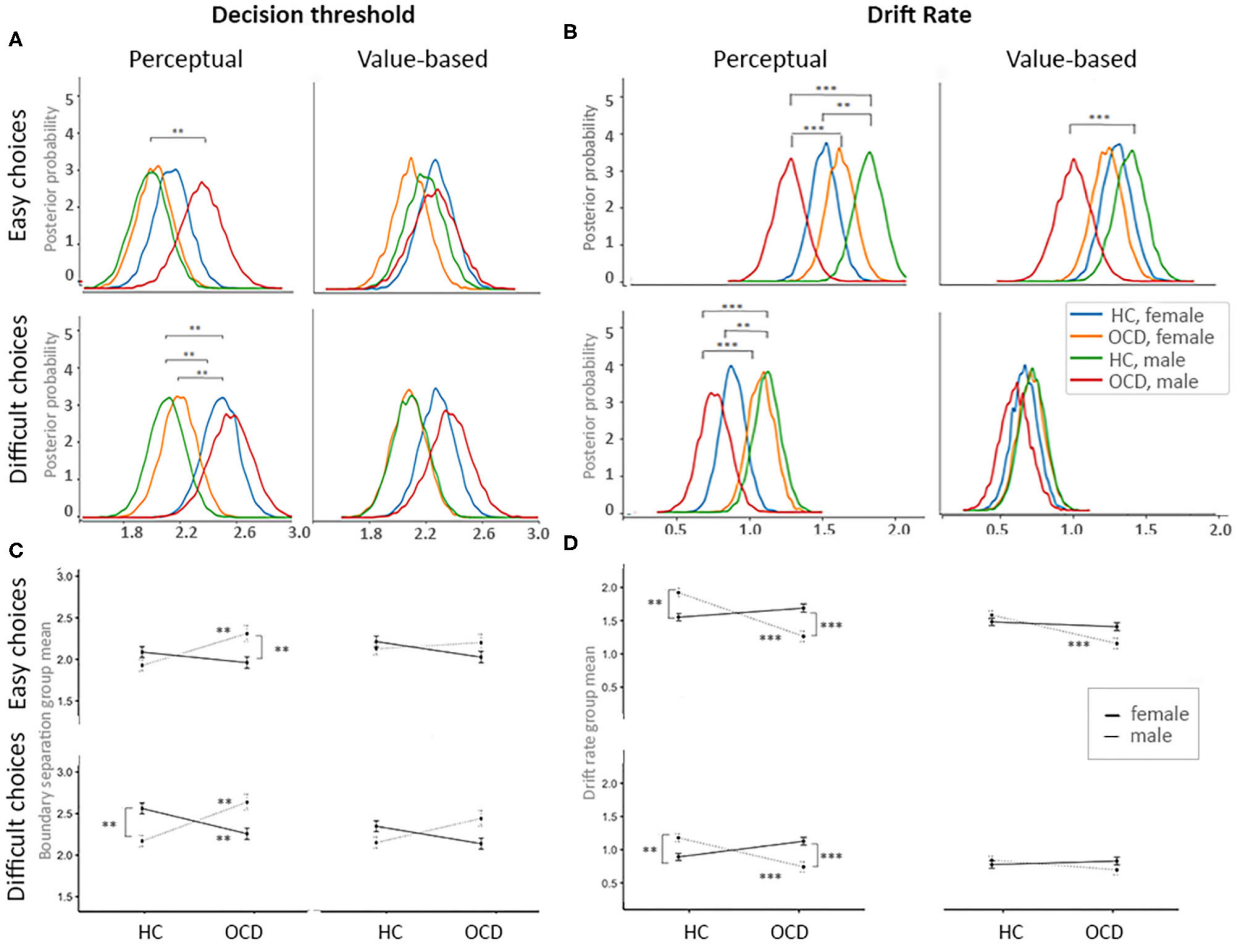
Effects of OCD diagnosis and gender on the process of evidence accumulation. **(A)** Posterior probability plots for decision threshold for each of four decision contexts across four groups of interests. **(B)** Posterior probability plots for the drift rates for each of four decision contexts across four groups of interests. **(C)** Mean plots for the decision thresholds for each of four decision contexts across four groups of interests. **(D)** Mean plots for the drift rates for each of four decision contexts across four groups of interests. Significance levels: **p = 0.05, ***p = 0.01.

OCD diagnosis affected both decision threshold and drift rate in males, but not in females (Figure 3). Specifically, in males with OCD the decision threshold was higher than in healthy males (see Figures 3A,C), indicating a more cautious decision style; this effect reached significance during perceptual decisions, both easy [*posterior* p (a_OCD, male_ > a_HC, male_) > 0.95] and difficult [*posterior* p (a_OCD, male_ > a_HC, male_) > 0.95]. We did not observe this effect in females. Also, in males with OCD, the drift rate was reduced as compared to healthy males [*posterior p* (v_HC, male_ > v_ocd, male_) > 0.99, Figures 3B,D]. During perceptual choices (both easy and difficult) and easy value-based choices, drift rate was significantly lower in males with OCD than in females with OCD, suggesting poorer quality of evidence accumulation. Increased decision thresholds and reduced drift rates have been previously reported in OCD by Banca, Vestergaard (17), using a different perceptual decision task. This previous study did not control for potential gender differences. Our results suggest that the reported effect is specific to males.

In a follow-up analysis, we employed Model 1 to examine the effect of OCD diagnosis on DDM parameters without accounting for gender differences (Supplementary Materials 3). In this analysis, no significant effects of OCD diagnosis on decision threshold were detected. The drift rate was lower in OCD during easy choices [both perceptual and value-based; *posterior p* (v_HC, male_ > v_ocd, male_) > 0.99]. Note that this last result is consistent with result reported by Banca, Vestergaard (17). We also employed Model 2 and examined the effect of gender in the pooled sample of individuals with OCD and healthy individuals. No gender effects on the DDM parameters were detected. These comparisons emphasize the importance of accounting for Gender x Diagnosis interactions in such analyses.

#### Flexible Adjustment to the Task Demands

**Figure 4:**
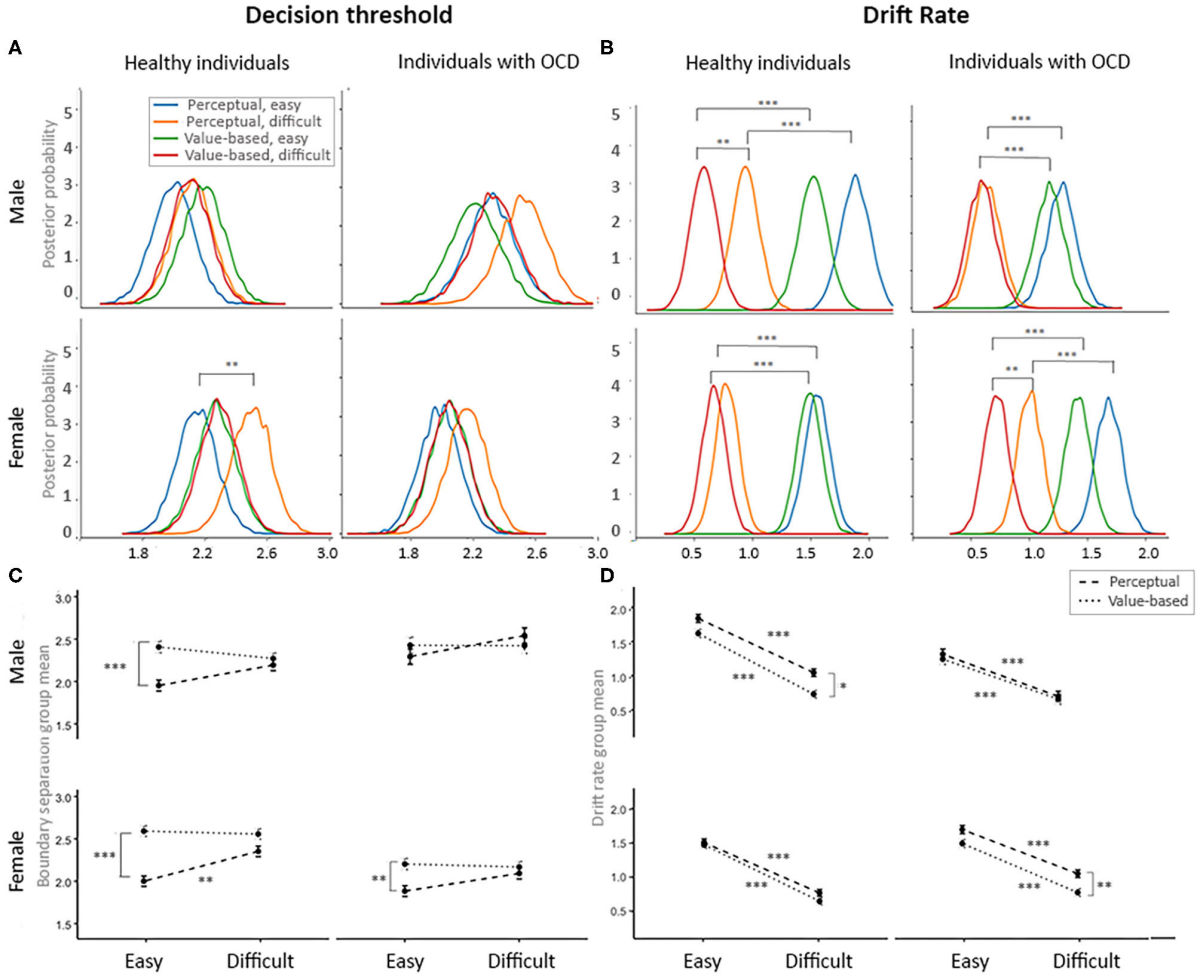
Flexible adjustment to the task demands by diagnostic and gender groups. **(A)** Posterior probability plots of the decision threshold for each of four groups of interest across four decision contexts. **(B)** Posterior probability plots for the drift rates for each of four groups of interest across four decision contexts. **(C)** Mean plots for the decision thresholds for each of four groups of interest across four decision contexts. **(D)** Mean plots for the drift rates for each of four groups of interest across four decision contexts. Significance levels: *p = 0.10, **p = 0.05, ***p = 0.01.

Finally, we examined how the study participants adjusted to task demands across experimental conditions (Figure 4).

Contrary to prior studies (25, 27), we found that during easy trials, healthy males and females and females with OCD accumulated less information during perceptual than during value-based decisions *posterior p* [HC males and HC females: *posterior p* (a value-based, easy > a perceptual, easy) > 0.99; OCD females: *posterior p* (a value-based, easy > a perceptual, easy) > 0.95]. Males with OCD accumulated the same amount of information during PDM and VDM trials. Note that, in contrast to past studies, we controlled for age and IQ of our participants, and we used exactly the same stimuli in PDM and VDM choices. We also observed a trend toward accumulation of more information during difficult perceptual choices compared to easy perceptual choices in males and females with OCD and in healthy males [*posterior p* (a perceptual, easy < a perceptual, difficult) > 0.90]; in healthy females this effect reached significance [*posterior p* (a perceptual, easy < a perceptual, difficult) > 0.95; see Figures 4A,C].

As expected, study participants were more efficient in processing evidence during easy choices (high drift rate, corresponding to high signal-to-noise ratio) than during difficult choices (low drift rate) during both perceptual and value-based choices [(*posterior p* (v easy > v difficult) > 0.99) Figures 4B,D]. This change in the drift rate in response to choice difficulty was reduced for males with OCD during VDM trials [*posterior p* (v easy > v difficult) > 0.95]. Consistent with prior studies (25, 27), healthy males were more efficient in processing evidence during easy perceptual trials than during easy value-based trials [*posterior p* (v perceptual, easy > v value-based, easy) > 0.95]. We did not observe this effect other participants groups (Figures 4B,D).

Overall, for males with OCD our analyses failed to detect significant changes in DDM parameters OCD across conditions (VDM vs. PDM, easy vs. difficult trials) more often than for other groups of interest. Decision thresholds remained at higher levels than for other groups across conditions; this difference was strongest during easy choices. Drift rates remained at lower levels in males with OCD than in other groups, across conditions; this difference was stronger in perceptual and in easy choices (Figure 4). This may indicate reduced behavioral flexibility in this group. This result is consistent to findings by Riesel, Kathmann (65), who reported that DDM parameters adjusted to experimental manipulations (instructions to prioritize either accuracy or speed) less in OCD than in healthy individuals; note, however, that they did not control for gender effects.

## Discussion

We introduce a novel decision task that allows characterization of the process of evidence accumulation across easy and difficult choices and across perceptual and value-based judgments. Importantly, the same neutral stimuli – grayscale images – were used across conditions, and task difficulty and “liking” of the stimuli was based on individual ratings and was balanced across subjects and conditions. This allows us to examine how the evidence accumulation process adjusts in response to task demands in healthy and clinical populations, while controlling for several potential confounds. Here, we demonstrate that the evidence accumulation process adjusts in response to task demands, and that this adjustment is altered in individuals with OCD – but not equally across genders. We find that males with OCD, but not females, accumulated more information (i.e., were more cautious) and were less effective in evidence accumulation than age- and IQ-matched healthy males. We also find that males with OCD, but not females, were less likely to adjust a process of evidence accumulation across decision contexts.

Sexual dimorphism in how OCD affects evidence accumulation is a novel finding but not entirely surprising. Individuals with a diagnosis of OCD are markedly heterogeneous (29–32). This heterogeneity is not well-understood; it complicates diagnosis and treatment selection. Gender differences in OCD-associated impairments in executive functions remain understudied (33–35). However, reviews of this literature have progressed over the decades from dismissing the possibility of gender effects in OCD (36) to acknowledging growing evidence for their importance (29, 35, 37, 38). For instance, females with OCD exhibit greater comorbidity with impulse-control disorders (38), which suggests gender differences in decision formation in OCD. Recent meta-analysis suggests that the proportion of females in samples of study participants may moderate estimates of some commonly reported neuropsychological impairments in OCD (45). However, laboratory studies of OCD, both behavioral and imaging, do not often examine whether gender modulates impairments in decision making in OCD; indeed, examining the effect of gender is often impossible in the small samples typically used in laboratory-based behavioral studies, due to limited statistical power. Hierarchical parameter estimation in drift diffusion models of choice improves power in estimation of both group-level tendencies and individual variation in latent cognitive processes as compared to non-hierarchical analyses of behavioral data (8, 10), and thus allows us to address gender differences in a sample of relatively modest size (*N* = 58).

We find that OCD affects evidence accumulation during choice, but only in males. First, males with OCD had higher decision thresholds than healthy males; this effect was stronger during perceptual judgments. We did not observe this effect of OCD in females. The decision threshold parameter can be interpreted as a measure of impulsivity during choice. Impulsivity is a complex trait and can be conceptualized and quantified in different ways. For instance, prior studies divided impulsivity into decisional and motor subtypes (62). They suggested that decisional impulsivity includes reflection impulsivity (the amount of information gathered before taking a decision, i.e., the decision thresholds) and delay discounting (a measure of the subjective discounting of a delayed reward). Motor impulsivity includes reduced motor response inhibition and premature or anticipatory responding (64). We find that reduced reflection impulsivity in OCD, which has been reported before (17), is specific to males; this result is potentially consistent with prior epidemiological finding that impulse-control disorders are seen less often in males than in females with OCD (38). Note that none of our study participants had impulse control disorder, so differential comorbidity cannot explain our results.

Second, males with OCD were less effective in processing of information than healthy males; this effect was also more pronounced during perceptual judgements and easy decisions. Moreover, while healthy males were more efficient in dealing with perceptual information than healthy females, males with OCD were less efficient than females with OCD. Prior studies employed the moving dots task to probe OCD-associated impairments in evidence accumulation during perceptual judgements (17, 66), however, they did not control for potential gender effects. These studies reported increased decision thresholds in OCD, especially under high uncertainty (i.e., during more difficult decisions) and reduced drift rate in OCD under low uncertainty (i.e., during easier decisions) (17). When we did not control for gender effects (Model #1) we also found reduced drift rate in OCD during easy choices; but other effects of OCD diagnosis were attenuated (reflecting omitted-variable bias). Prior studies of evidence accumulation in OCD suggested that both increased decision thresholds and decreased drift rates might contribute to the excessive doubt and indecisiveness that are commonly observed in OCD. Our results suggest that this effect might be gender specific.

Third, a process of evidence accumulation adjusted in response to experimental manipulations (perceptual vs. value-based and easy vs. difficult trials) in males with OCD less than it did in other groups of participants. A similar effect was previously reported for OCD by Riesel, Kathmann (65), who did not control for gender effects; it is consistent with the reduced cognitive and behavioral flexibility commonly observed in OCD. However, our results suggest that this effect might be gender specific as well. The ability to flexibly adjust how much information needs to be accumulated prior to making a choice in response to task demands has been linked to the functioning of the subthalamic nucleus [STN; (67)]. Thus, our findings suggest that the STN, and associated basal ganglia circuits, may be more (or differently) dysregulated in males than in females with OCD. Further studies are needed to test this hypothesis.

It is important to note that the moving dots task, the most commonly used task to probe evidence accumulation in OCD, requires participants to accumulate evidence in the presence of uncertainty. In contrast, the stimuli in the PVDM task are certain. Thus, it the two paradigms may probe different OCD-associated impairments. Future studies should examine how evidence accumulation in OCD adjusts across all three contexts: perceptual judgement under uncertainty (RDM) and perceptual and value-based judgment under certainty (PVDM).

Other tasks have been used to compare perceptual and value-based decision formation in the general population. For instance, in one design, during perceptual choice, participants were asked to judge the proportion of white and black marbles on the screen; during value-based choice, participants were asked to assign a positive value to white marbles and a negative value to black marbles, and choose between a gamble represented by the collection of the marbles on the screen and the reference 50–50 gamble (27). This design uses the same stimuli in both conditions, however, in the value-based condition it also introduces uncertainty (and individual risk attitudes) that is not present in a perceptual condition, complicating interpretation. Another design uses images of snacks as stimuli in both conditions (25). During perceptual judgments, participants are asked “how much (in percent) they thought the food item was covering the black background within the white square,” or “which of the presented food snacks covers more of the black background;” during value-based judgements, participants were asked to decide “how much they wanted to eat the presented food snack at the end of the experiment,” or “which of the presented food snack they wanted to eat at the end of the experiment.” After the experiment, subjects were required to stay in the room with the experimenter while eating the food item that they chose in a randomly selected trial from the value-based condition. This last component is an important part of the design since it generates incentives to provide accurate ratings. However, including such incentives in the study of clinical populations, such as OCD, may raise complications, as some subjects may have complicated attitudes toward food (e.g., contamination concerns, comorbid eating disorders) or toward eating in the presence of the experimenter. Neutral grayscale images as stimuli and incentives, which we employ here, are less likely to interact with symptomatology and thus may be more suitable for research in clinical populations. Using incentive-based designs is arguably an advantageous approach and is often used by experimental and behavioral economics. This approach generally improves participants' engagement and allows estimating behavior-based measures more accurately, since these measures are based on consequential choices (68). Whether incentives significantly affect choices in our design is an empirical question and beyond the scope of this study.

We call for future research to incorporate gender-balanced samples and account for potential gender effects in tests of how OCD may impact evidence accumulation across diverse decision contexts. We also suggest that future studies transdiagnostically investigate potential gender differences in evidence accumulation; it is possible that general distress in psychopathology affects evidence accumulation differently in males and females – that is, that the differences we observe are not specific to OCD. This possibility is consistent with results reported by Lighthall, Sakaki (69) that gender differences in decision formation were present in stressed participants but not controls, and that stress led to greater reward collection and faster decision speed in males but less reward collection and slower decision speed in females. It has been suggested that reduced efficiency of evidence accumulation can serve as a transdiagnostic marker of vulnerability to psychopathology (70). Our results suggest that this transdiagnostic marker could be gender dimorphic. Carefully designed laboratory studies that include participants across diagnostic groups that are well-powered and gender balanced may distinguish between diagnosis-specific and transdiagnostic effects (e.g., doubt, reduced efficiency in evidence accumulation), as well as effects of general distress on evidence accumulation and decision formation, and examine the possibility of gender dimorphism for all of them.

Our four samples (healthy females, healthy males, females with OCD and males with OCD) were well-matched on demographic characteristics that might contribute to differences in decision making, such as age, education, and income. Our OCD participants had somewhat lower IQ that healthy participants, on average. Since both prior research and our data indicates that IQ can affect both the decision threshold and the drift rate, this difference could potentially confound our results. To address this problem, we included both age and IQ as covariates in the computational model. Even if this remedy was not sufficient, the difference in IQ is unlikely to account for observed sex-dimorphic effects of OCD on the decision threshold. Our findings warrant future investigations in larger and better-balanced samples.

Similarly, the small difference in OCD severity between males and females with OCD in our sample might bias our results. But this, too, is unlikely to explain the presence of effects of OCD diagnosis on decision threshold and drift rates in males but not females, who had somewhat more severe OCD symptoms. OCD symptom dimensions were similarly represented between males and females, arguing against symptom type as an explanation for the observed gender differences.

Overall, our findings contribute to a sparse literature on gender-related heterogeneity in obsessive-compulsive disorder. Clinicians should be mindful of the possibility of gender-specific impairments in OCD, and researchers should power future studies adequately to rigorously assess gender effects both in diagnosis specific and transdiagnostic investigations.

## Data Availability Statement

The raw data supporting the conclusions of this article will be made available by the authors, without undue reservation.

## Ethics Statement

The studies involving human participants were reviewed and approved by Yale University Human Subjects Committee. The patients/participants provided their written informed consent to participate in this study.

## Author Contributions

HP and CP conceived of the presented idea and supervised the work. HP designed the behavioral task and the experiment. XM, AM, and HP collected the data. XM and AM pre-processed the data. XM and HP analyzed the data. All authors contributed to the article and approved the submitted version.

## Conflict of Interest

The authors declare that the research was conducted in the absence of any commercial or financial relationships that could be construed as a potential conflict of interest.

## Publisher's Note

All claims expressed in this article are solely those of the authors and do not necessarily represent those of their affiliated organizations, or those of the publisher, the editors and the reviewers. Any product that may be evaluated in this article, or claim that may be made by its manufacturer, is not guaranteed or endorsed by the publisher.
