# Supplementary material for: OCD Influences Evidence Accumulation During Decision Making in Males but Not Females During Perceptual and Value-Driven Choice
Source: Front Psychiatry. 2021 Jul 29;12:687680. doi: 10.3389/fpsyt.2021.687680 (PMC8358201; doi:10.3389/fpsyt.2021.687680)
Supplement: Supplementary file 1 [file Data_Sheet_1.docx]

# *Supplementary Appendices*

# *for*

# *OCD influences evidence accumulation during decision making in males but not females during perceptual and value-driven choice.*

**S1. Sample description**

|  | | **Healthy Individuals** | | | | | | | **Individuals with OCD** | | | | | | | **OCD vs. HC** | | | | **Across 4 groups** | | | |  |
| --- | --- | --- | --- | --- | --- | --- | --- | --- | --- | --- | --- | --- | --- | --- | --- | --- | --- | --- | --- | --- | --- | --- | --- | --- |
|  | | **Males** | | **Females** | | **Pooled** | **HC: males vs. females** | | **Males** | **Females** | **Pooled** | **OCD: males vs. females** | | | |  |  |  |  |  |  |  |  |  |
|  |  |  |  |  |  |  | test statistics | p-value |  |  |  | test statistics | | p-value | | test statistics | | p-value | | test statistics | | p-value | |  |
| *Demographics* | | | | | |  |  |  |  |  |  |  | |  | |  | |  | |  | |  | |  |
|  | | | **N = 16** | | **N = 15** | **N = 31** |  |  | **N = 11** | **N = 16** | **N = 27** |  | |  | |  | |  | |  | |  | |  |
|  | | mean (SD) | | | | | t(29) | | mean (SD) | | | t(25) | | | | t(56) | | | | F(3, 54) | | | |  |
| Age | | 29.6 (10.1) | | 27.5 (11.4) | | 28.5 (10.6) | -0.5 | 0.6 | 32.8 (13) | 29.1 (9.5) | 30.6 (11) | | 0.9 | | 0.4 | | -0.7 | | 0.5 | | 0.52 | | 0.67 | |
| IQ | | 112 (13.3) | | 114 (12.2) | | 113 (12.7) | 0.5 | 0.6 | 107 (14) | 105 (13.7) | 106 (13.7) | | -0.4 | | 0.7 | | **2** | | **0.03** | | 1.68 | | 0.18 | |
|  | | median | | | | |  | | median | | |  | | | |  | | | |  | | | |  |
| Education | | 5  (college graduate) | | 5  (college graduate) | | 5  (college graduate) | *Χ^2^(4, 29) = 2* | 0.7 | 4  (some college) | 4.5  (some college & college graduate) | 4  (some college) | *Χ^2^(4, 26) =* 2 | | 0.7 | | *Χ^2^(4, 55) =* 6 | | 0.2 | | *Χ^2^(10, 55) = 10* | | 0.6 | |  |
| Income | | 4  ($35,000-$49,999) | | 5  ($50,000-$74,999) | | 5  ($50,000-$74,999) | *Χ^2^(7, 29) =* 8 | 0.3 | 5  ($50,000-$74,999) | 4  ($35,000-$49,999) | 4  ($35,000-$49,999) | *Χ^2^(7, 26) = 7* | | 0.5 | | *Χ^2^(7, 55) = 8* | | 0.3 | | *Χ^2^(20, 55) = 2*0 | | 0.5 | |  |
| *Clinical symptoms* | | | |  | |  |  |  |  |  |  |  | |  | |  | |  | |  | |  | |  |
|  | **N = 15** | | | **N = 14** | | **N = 29** |  |  | **N = 10** | **N = 16** | **N = 26** |  | |  | |  | |  | |  | |  | |  |
|  | | mean (SD) | | | | | t(27) | | mean (SD) | | | t(24) | | | |  | | | | F(3, 51) | | | |  |
| YBOCS | | - | | - | | - | - | - | 21.8 (3.5) | 24.6 (5.5) | 23.5 (4.9) | 1 | | 0.2 | | - | | - | | - | | - | |  |
| Obsessions | | - | | - | | - | - | - | 10.5 (1.8) | 11.9 (2.9) | 11.3 (2.6) | 1 | | 0.2 | | - | | - | | - | | - | |  |
| Compulsions | | - | | - | | - | - | - | 11.3 (2.1) | 12.7 (2.9) | 12.2 (2.6) | 1 | | 0.3 | | - | | - | | - | | - | |  |
| BDI - II | | 6.9 (7.3) | | 4.2 (4.0) | | 5.6 (6.0) | t(22) = -1† | 0.2 | 10.8 (12.8) | 13.3 (12.9) | 12.4 (12.6) | 0.5 | | 0.6 | | **t(35) = -2**† | | **0.2** | | 2.2 | | 0.10 | |  |
| DOCS | | 7.9 (9.4) | | 5.8 (5.6) | | 6.9 (7.8) | -0.7 | 0.5 | 22.0 (9.4) | 27.0 (11.5) | 25.1 (10.8) | 1 | | 0.3 | | **t(53) = -7** | | **< 0.01** | | 18.1 | | **< 0.01** | |  |
| OCI-R | | 6.9 (9.1) | | 5.9 (5.4) | | 6.4 (7.4) | -0.4 | 0.7 | **18.5 (7.5)** | **27.7 (10.1)** | **24.0 (10.1)** | **2** | | **0.02** | | **t(52) = -7** | | **< 0.01** | | 22.3 | | **< 0.01** | |  |
| Washing | | 0.4 (0.8) | | 1.0 (1.2) | | 0.7 (1.1) | 2 | 0.1 | 3.8 (2.9) | 6.4 (3.9) | 5.4 (3.7) | 2 | | 0.08 | | **t(27) = -6**† | | **< 0.01** | | 17.7 | | **< 0.01** | |  |
| Checking | | 1.7 (2.7) | | 0.5 (0.9) | | 1.1 (2.1) | t(17) = -2† | 0.1 | 4.0 (3.0) | 5.0 (3.2) | 4.6 (3.1) | 0.8 | | 0.40 | | **t(53) = -5** | | **< 0.01** | | 9.0 | | **< 0.01** | |  |
| Obsessing | | 0.7 (0.9) | | 0.4 (1.1) | | 0.5 (1.0) | -0.8 | 0.4 | 3.7 (3.5) | 3.2 (2.5) | 3.4 (2.9) | -0.4 | | 0.7 | | **t(30) = -5**† | | **< 0.01** | | 8.5 | | **< 0.01** | |  |
| Neutralizing | | 0.7 (1.4) | | 0.2 (0.4) | | 0.4 (1.0) | t(17) = -1† | 0.2 | 1.2 (1.6) | 3.2 (3.4) | 2.4 (3.0) | t(22) = 1.98† | | 0.06 | | **t(30) = -3**† | | **< 0.01** | | 6.0 | | **< 0.01** | |  |
| Ordering | | 1.7 (2.5) | | 2.2 (2.3) | | 2.0 (2.4) | 0.5 | 0.6 | 4.5 (3.3) | 6.4 (3.2) | 5.7 (3.4) | 1 | | 0.2 | | **t(53) = -5** | | **< 0.01** | | 8.9 | | **< 0.01** | |  |
| Hoarding | | 1.7 (2.3) | | 1.6 (1.7) | | 1.6 (2.4) | -0.1 | 0.9 | 1.3 (1.8) | 3.4 (3.3) | 2.6 (3.0) | 2 | | 0.08 | | t(43) = --1† | | = 0.2 | | 2.2 | | 0.22 | |  |
| BIS | | 63.4 (10.0) | | 60.7 (6.4) | | 62.2 (8.4) | -.9 | 0.37 | 56.5 (9.2) | 64.9 (11.8) | 61.7 (11.4) | 1.9 | | 0.07 | | t(53) = 0.18 | | = 0.86 | | 1.77 | | 0.17 | |  |

**Note:** In **bold** are significant differences between groups. † - assumption of equality of variance was violated (i.e. Levine’s test p-value < 0.05), statistics was reported from Welch t test (i.e. equality of variance was not assumed).

**Table S1** illustrates that four groups of interest – healthy males, healthy females, males with OCD and females with OCD – were matched on age, education, and income. Healthy participants on average scored approximately 8 point higher on IQ test than did participants with OCD.

Three of 57 participants who completed the behavioral task did not complete selected self-report measures (BDI-II, DOCS, and OCI-R): one healthy male, one healthy female, and one male with OCD. Thus, clinical symptoms are reported for 54 participants. Females with OCD reported more severe symptoms than males with OCD, and for OCI-R this difference reached nominal statistical significance at p = 0.03.

We conducted secondary analyses to examine whether OCD dimensions were distributed evenly between gender. We performed a repeated measures ANOVA with the repeated measures OCD dimensions, and the between-subject factor gender. This analysis revealed that the main effect of *gender* on OCD severity was significant at p = 0.016 (F(5,20) = 3.697), but the interaction term *OCD dimensions x gender* was not significant at p = 0.11 (F(5,20) = 2.118). Therefore, the gender -related differences that we report in the paper are unlikely to be driven by group differences in OCD dimensions.

**S2. Model selection**

We fit reaction time and accuracy to 3 alternative models:

Model 1 (DIC_Dx_ = 41090): *a* ~ trial type, choice difficulty, **Dx**; *v* ~ trial type, choice difficulty, **Dx**; *τ* ~ trial type, choice difficulty, **Dx**; *z*;

Model 2 (DIC_Gender_ = 41090): *a* ~ trial type, choice difficulty, **gender**; *v* ~ trial type, choice difficulty, **gender**; *τ* ~ trial type, choice difficulty, **gender**; *z*;

Model 3 (DIC_Dx x Gender_ = 41090): *a* ~ trial type, choice difficulty, **Dx, gender**; *v* ~ trial type, choice difficulty, **Dx, gender**; *τ* ~ trial type, choice difficulty, **Dx, gender**; *z*.

Deviance information criteria (DIC) for the three models were similar, with Model 3 slightly lower. Posterior predictive plots (PPP) of reaction time distribution for all healthy participants and all OCD participants (generated separately), were also similar across three models, and all indicated a good fit of models to data. However, PPP that contrasted the predicted data with the actual data for males and females separately, indicated that (1) the distributions of reaction time differed across Dx x Gender groups, and (2) the model fit for females separately was better (see figure S1 below). Thus Model 3 was selected.


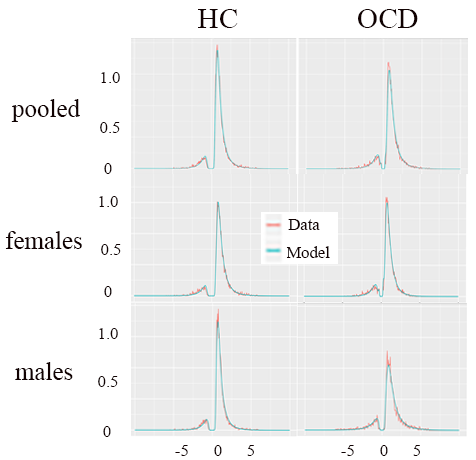


**Figure S2.** Posterior predictive plots for (A) all healthy participants and all OCD participants using Model 1, and (B) for healthy females, healthy males, females with OCD, and males with OCD, using Model 3.

**S3. Follow up analyses – fitting data to Model 1 that does not allow gender differences**

Since in our sample effect of OCD on evidence accumulation (especially during perceptual decisions) is different in males and females, we conducted a follow-up analysis. We employed Model 1, which does not incorporate gender (see S2), and examined the effect of OCD diagnosis on DDM parameters without accounting for gender differences. No significant effects of OCD diagnosis on decision threshold were detected. The drift rate was lower in OCD during easy choices (both perceptual and value-based). **Figure S3** depicts posterior probability plots for healthy participants and participants with OCD across four decision contexts.


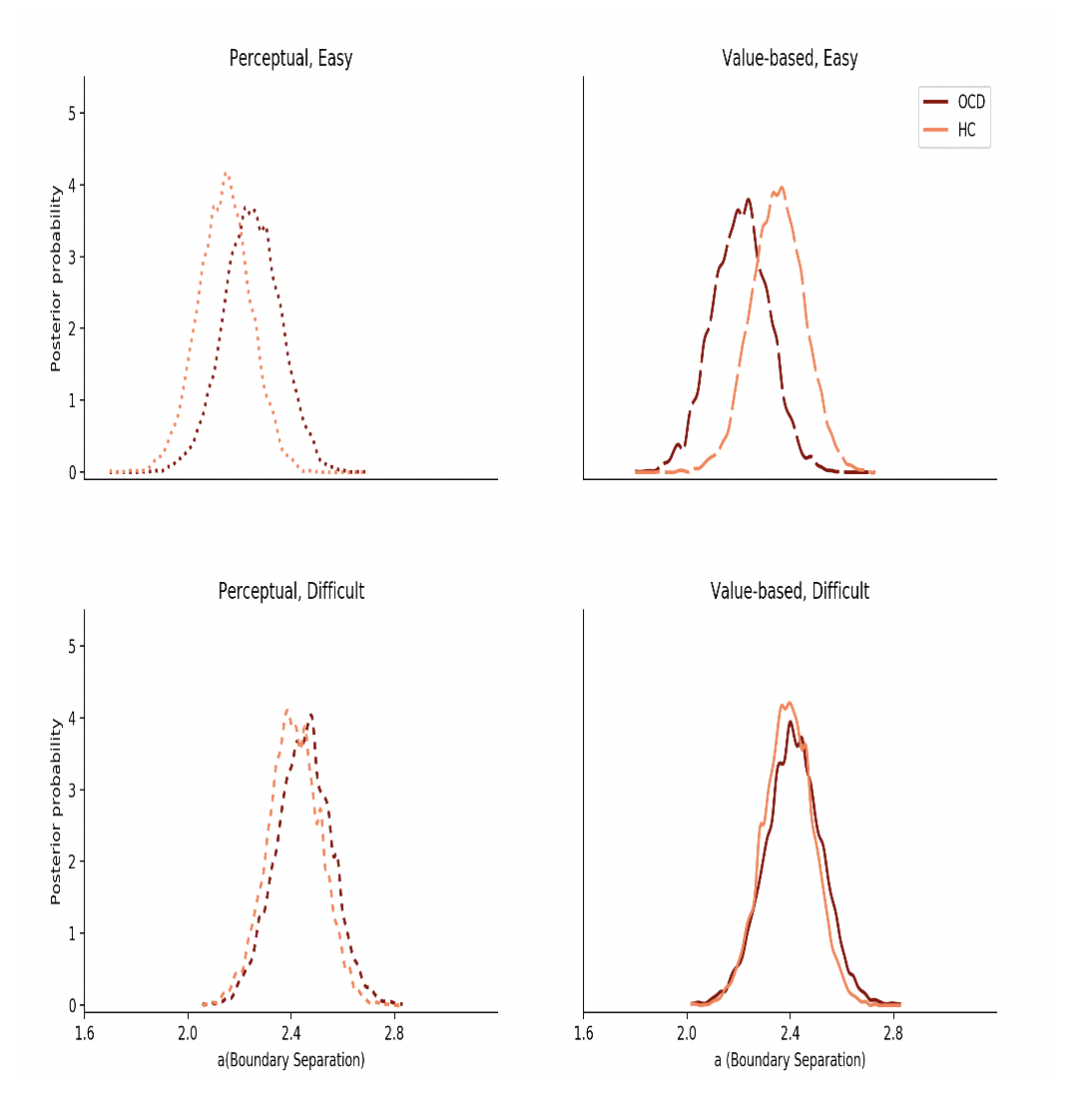


**Figure S3A.** Posterior probability plots for the decision threshold based on Model 1.


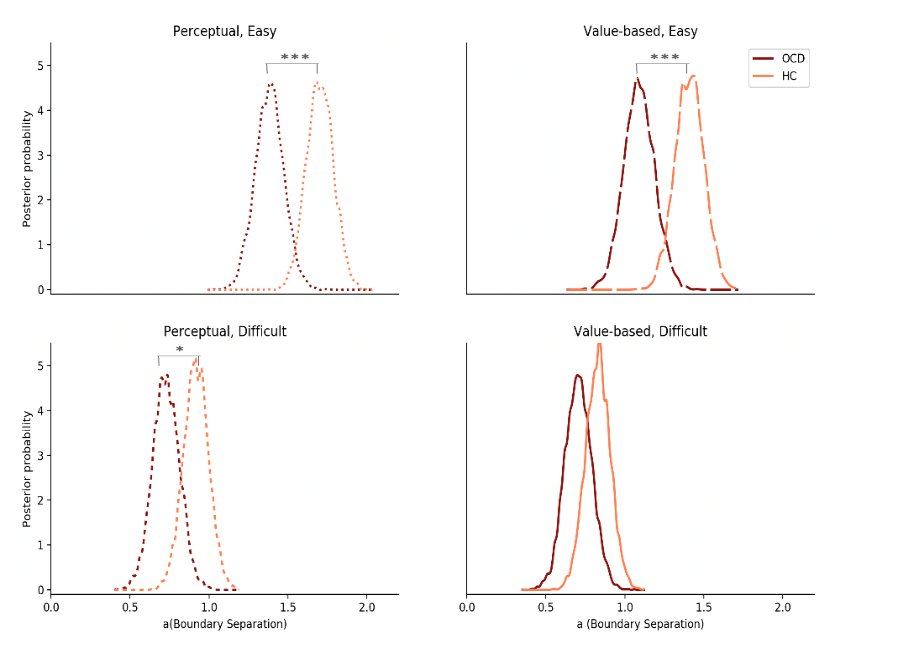


**Figure S3B.** Posterior probability plots for the drift rate based on Model 1. Note: ^***^- significance level is p < 0.01.

**S4. Follow up analyses – fitting data to Model 2 that does allow gender differences**

We also employed Model 2, which does incorporate gender, and examined the effect of gender on DDM parameters without accounting for OCD diagnosis. No significant effects of gender on decision threshold or drift rate were detected. **Figure S4** depicts posterior probability plots for female and male participants across four decision contexts.


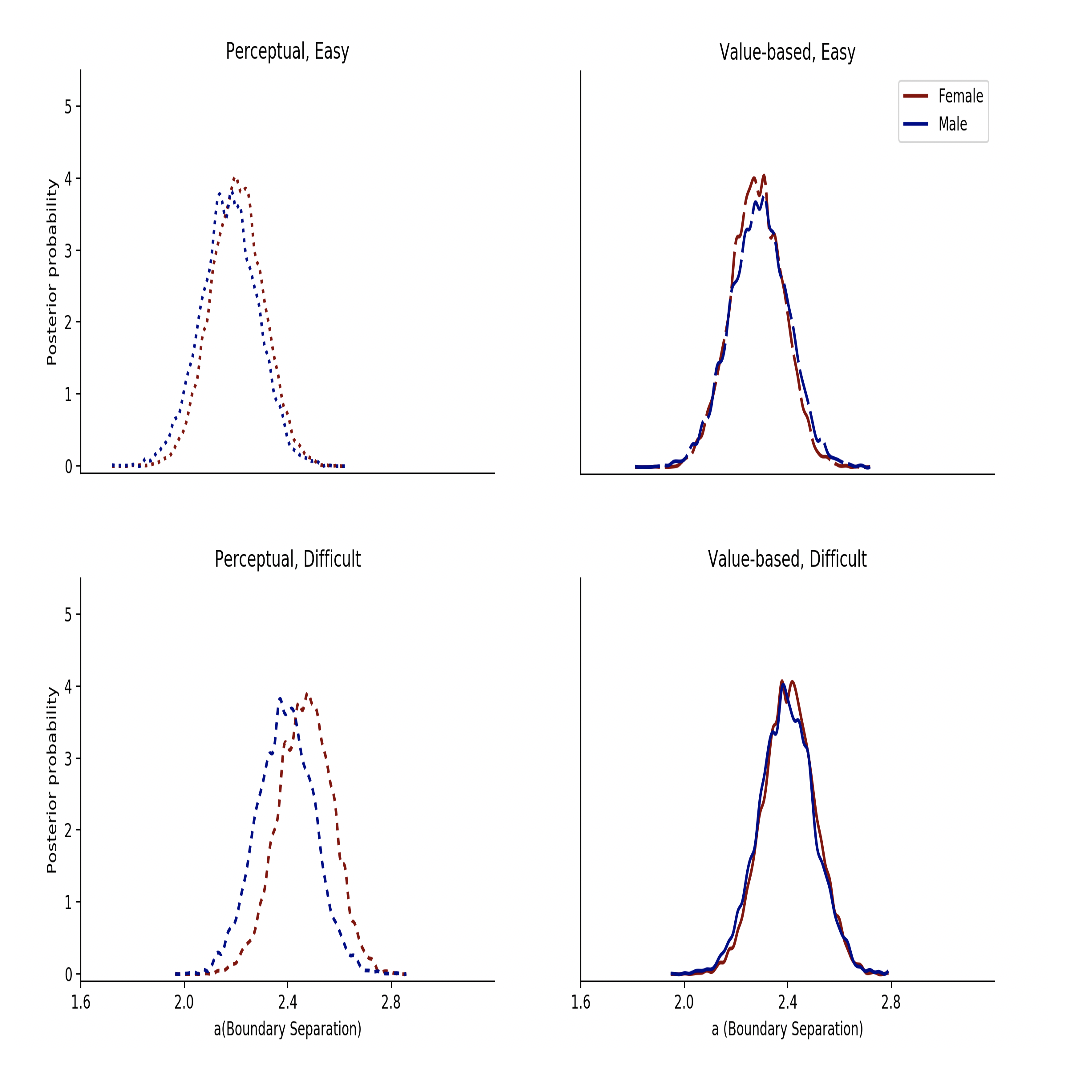


**Figure S4A.** Posterior probability plots for the decision threshold based on Model 2.


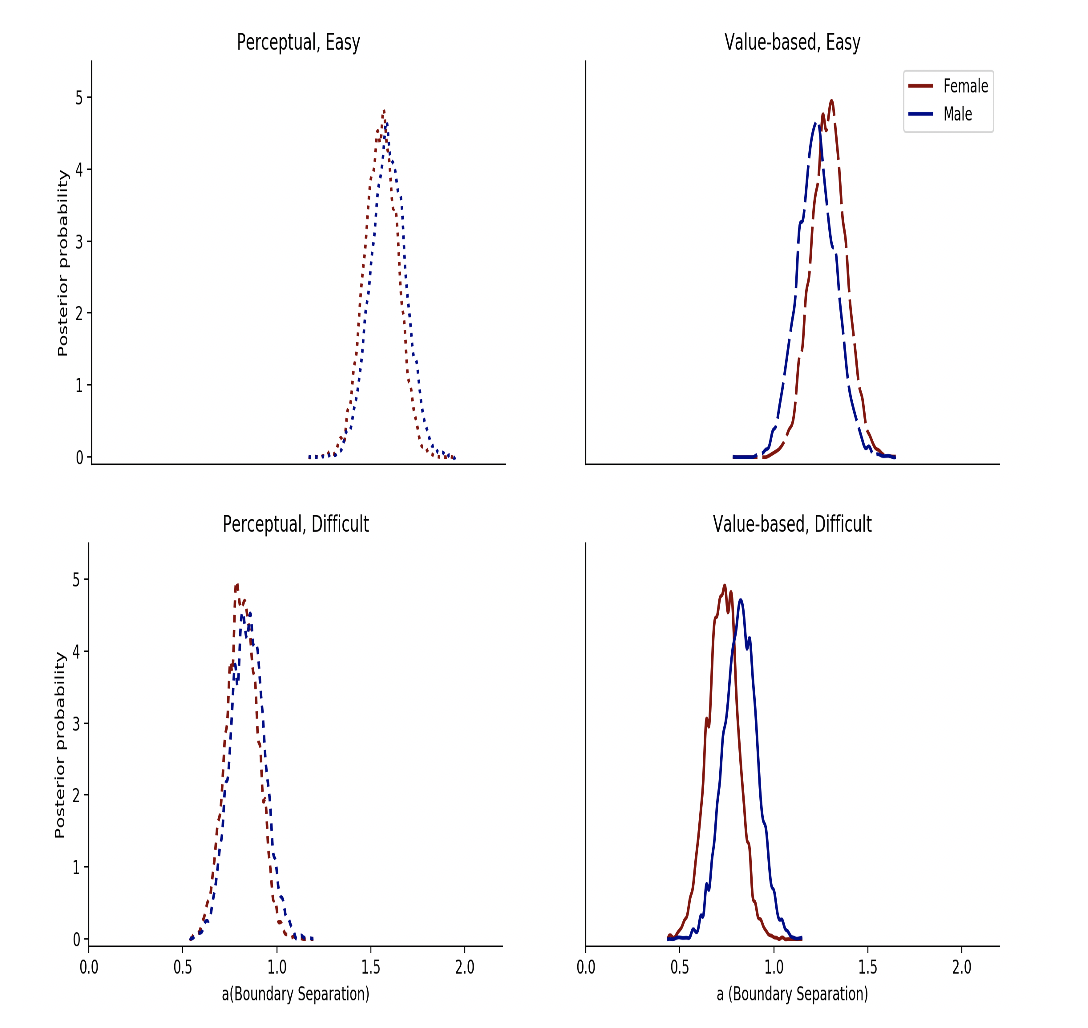


**Figure S4B.** Posterior probability plots for the drift rate based on Model 2.

Note: ^***^- significance level is p < 0.01.
